# Effective capnography training in the ICU using the 'hats and caps' training tool

**DOI:** 10.1186/cc14344

**Published:** 2015-03-16

**Authors:** CA Lobo, FE Kelly, S Steynberg, G Thomas, C Pope, M Eveleigh, M Charlton, TM Cook

**Affiliations:** 1 Royal United Hospital, Bath, UK

## Introduction

Failure to use or correctly interpret capnography in patients dependent on an artificial airway in ICUs was thought to have contributed to 74% of ICU airway-related deaths in the NAP4 study [1]. However, capnography is only of value if those using it can interpret it correctly, with recommendations for training all ICU staff in capnography [1,2]. A recent UK survey identified that only 48% of ICUs have trained all staff in capnography interpretation (TM Cook, personal communication). In this study, we used a capnography teaching aid ('hats and caps') to educate all ICU staff during a 1-month period, and evaluated its effectiveness.

## Methods

**Figure 1:**
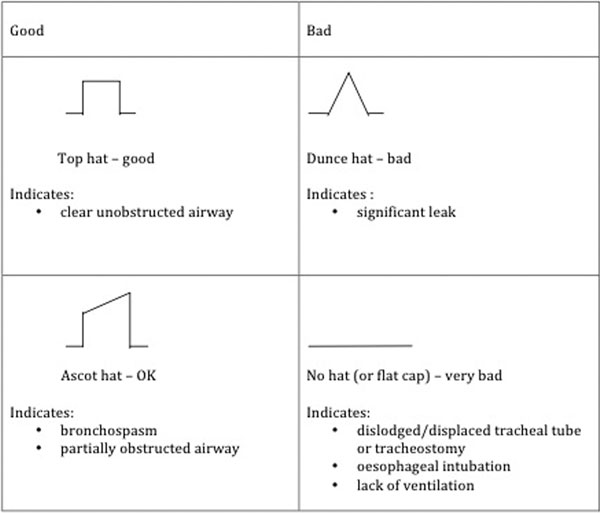
**'Hats and caps' capnography training guide**.

'Hats and caps' was devised on our ICU [3] and used for the training: this teaches that capnography traces on the left signify the airway is functional, in contrast to the traces on the right which indicate immediate attention is required (Figure 1). This was presented to staff working on the ICU in individual bedside teaching sessions with feedback obtained and evaluated.

## Results

We delivered teaching sessions to 100% (9/9) of junior doctors, 100% (71/71) of nursing staff and other health professionals. We obtained feedback from 90% (76/84), showing an improvement in understanding of capnography from 73% of respondents to 100%, with 87% reporting that the teaching aid made capnography interpretation much easier. All felt the training would improve patient safety, and 97% felt it would be worthwhile training in other ICUs.

## Conclusion

Use of 'hats and caps' enabled delivery of short bedside teaching sessions to clinical staff in ICU during everyday work. Feedback shows a marked improvement in confidence around capnography interpretation. It may have value in other ICUs to improve staff understanding of capnography and improve patient safety.
